# Effects of community health worker-based interventions on physical activity levels in children: a systematic review

**DOI:** 10.1590/1984-0462/2022/40/2020232

**Published:** 2021-10-25

**Authors:** Paulo Henrique Guerra, Rafael Silvestre, Tiago Henrique Toldo de Mello, Ana Luiza Babo Sedlacek Carvalho, Filipe Ferreira da Costa, Alex Antonio Florindo

**Affiliations:** a Universidade Federal da Fronteira Sul, Chapecó, SC, Brazil.; b Universidade Federal da Paraíba, João Pessoa, PB, Brazil.; c Universidade de São Paulo, São Paulo, SP, Brazil.

**Keywords:** Motor activity, Community health workers, Child, Adolescent, Systematic review, Atividade física, Agentes comunitários de saúde, Criança, Adolescente, Revisão sistemática

## Abstract

**Objective:**

To identify the strategies and effects of interventions carried out by community health workers (CHW) on physical activity (PA) levels in children and adolescents.

**Data source:**

In August 2020, a systematic review, designed according to the PRISMA checklist items, was conducted by searches in seven electronic databases and in reference lists. Original studies were searched without restriction with regard to year of publication; they were written in Spanish, English or Portuguese and examined interventions implemented by CHW, involving theoretical and/or practical contents of PA, with a focus on children and/or adolescents between three and 19 years of age.

**Data synthesis:**

Of the 2,321 studies initially retrieved, eight were included, targeting samples with more specific characteristics (e.g., clinical, ethnic and/or socioeconomic). In all studies, CHW were trained to lead educational activities. In three non-controlled trials, positive results were observed, involving indicators such as moderate and vigorous PA and physical inactivity reduction. Also, two positive results were found in reducing sedentary behavior.

**Conclusions:**

Even though most of the interventions included did not have a significant effect on increasing PA levels, the available findings reinforce the role of CHW as an important strategy for dialogue between health services and the most vulnerable communities, and they suggest a greater articulation of these professionals in the actions developed in the school context.

## RESUMO

**Objetivo:**

Identificar as estratégias e os efeitos de intervenções conduzidas por agentes comunitários de saúde (ACS) nos níveis de atividade física (AF) em crianças e adolescentes.

**Fontes de dados:**

Em agosto de 2020, uma revisão sistemática, delineada de acordo com os itens da lista de verificação PRISMA, foi desenvolvida por meio de buscas em sete bases de dados eletrônicas e em listas de referências. Foram procurados estudos originais, sem restrição quanto ao ano de sua publicação, escritos em espanhol, inglês e português, que desenvolveram intervenções implementadas por ACS envolvendo conteúdos teóricos e/ou práticas de AF com foco em crianças e/ou adolescentes na faixa entre os 3 e os 19 anos de idade.

**Síntese dos dados:**

Dos 2.321 estudos inicialmente avaliados, foram incluídos oito, direcionados a amostras com características mais específicas (clínicas, étnicas e/ou socioeconômicas). Em todos os estudos, ACS receberam treinamento prévio para a condução de atividades educativas. Em três ensaios não controlados foram observados resultados positivos envolvendo indicadores de AF moderadas, vigorosas e redução da inatividade física. Complementarmente, dois resultados positivos foram encontrados na redução do comportamento sedentário.

**Conclusões:**

Mesmo que a maior parte das intervenções incluídas não tenha apresentado efeitos significativos no aumento dos níveis de AF, a evidência disponível reforça o papel dos ACS como uma importante estratégia de diálogo entre os serviços de saúde e as comunidades mais vulneráveis e sugere maior articulação desses profissionais nas ações desenvolvidas no contexto escolar.

## INTRODUCTION

The scientific literature points out that the regular practice of physical activity (PA) positively impacts several domains of the lives of children and adolescents, with special emphasis on its relevance in health promotion,^1^ in the social aspect^2^ and in academic performance.^3^ However, even with all this contribution, high levels of insufficient PA are observed in different parts of the world,^4^ according to the recommendation of the daily practice of 60 minutes of moderate and vigorous PA.^5^

In view of the different negative health indicators that are associated with physical inactivity in the first decades of life,^6^ strategies for its promotion are recommended.^4^ However, in this age group, it is worth noting that the practice of PA is not only determined by individual issues.^7^ ^,^ ^8^ Recognizing that the contexts (economic, social and environmental) of an individual are also determinants for his/her health, various public policies have idealized the introduction of community health workers (CHW) in various countries, to expand access to care especially in places where there is little offer of services and specialized professionals.^9^

Previous studies suggest that interventions conducted by CHW show promising results in the improvement of various health indicators,^10^ ^,^ ^11^ including, for example, the increase in PA levels in populations of vulnerable adults. ^12^ Thus, in view of the benefits of PA to integral development of children, as well as the important role of CHW in care actions in various countries, this study sought to identify the effects of interventions conducted by CHW on the levels of PA in populations of children and/or adolescents. As a secondary objective, we also sought to evaluate the strategies used in interventions.

## METHOD

The present study was a systematic review of the literature and registered on the International Prospective Register of Systematic Reviews platform (CRD42019131832). Its design and writing were according to the items in the PRISMA checklist.^13^

As inclusion criteria, studies written in Spanish, English or Portuguese that developed interventions implemented by CHW involving theoretical contents and/or PA practices focused on children and/or adolescents in the age group between 3 and 19 years were sought. For the purposes of this work, CHW were defined as professionals not necessarily trained in health courses that received education/training on health topics to work (voluntary or paid) in their community or in populations that had characteristics (socioeconomic, cultural and ethnic, for example) that were close to them,^14^ regardless of whether they were working in the context of a health system or not.

No restrictions were imposed with regard to the designs of the intervention studies (such as the presence or absence of a control group, as well as the randomization between groups), research contexts (community, school, health unit), interventions with the presence of other themes of health (healthy eating, smoking, alcoholism), need for a link between the CHW and the local health system, nor regarding samples composed of children/adolescents with chronic non-communicable diseases (NCD) (example: obesity, hypertension and type 2 diabetes mellitus).

On August 19, 2020, the potential studies were retrieved through systematic searches in seven electronic databases - LILACS, PubMed, SciELO, Scopus, SPORTDiscus, Sports Medicine & Education Index and Web of Science -, based on the strategy developed for PubMed - (((((((((((((community health worker[Text Word]) OR chw[Text Word]) OR community health care worker[Text Word]) OR community health trainer[Text Word]) OR community case manager[Text Word]) OR community health aide[Text Word]) OR family planning personnel[Text Word]) OR lay health ­worker[Text Word]) OR community health officer[Text Word]) OR ­promoter[Text Word]) OR promoters[Text Word])) AND ((((((((physical activity[Text Word]) OR physical ­education[Text Word]) OR sports[Text Word]) OR exercise[Text Word]) OR ­walk*[Text Word]) OR run*[Text Word]) - and through manual searches in the reference lists of studies evaluated by their full texts. There was no prior limit imposed for the years of publication of the studies. The document that details the systematic searches used in each database can be obtained by contacting the corresponding author.

The procedures for evaluating titles and abstracts, full texts and data extraction were performed by two researchers (RS and TM), independently, with the help of a third researcher (PG). The extraction of the original data was done on an electronic spreadsheet, which was organized according to: descriptive information (research location, year of collection, sample size, age group, special characteristics of the sample and primary outcomes of the interventions), methods (characteristics and CHW actions, intervention protocol, PA assessment instruments and indicators) and results of interventions in PA indicators (magnitudes and p-values). Even though it was not the focus of the present study and therefore not addressed in systematic searches, information related to sedentary behavior (SB) (evaluation methods and results) was also collected and organized from the moment its occurrence was noticed in all studies included. In the current understanding, PA and SB represent different behaviors, based on the energy expenditure employed in each of them; SB represents activities with low energy expenditure and a body posture in which large groups of skeletal muscles have very little or no overload - usually performed with the body sitting -, and PA is classified into three categories, according to their energy demand: light (walking slowly and climbing stairs, for example), moderate (brisk walking and trot) and vigorous (competitive sports).^15^ The descriptive synthesis of the studies included was prepared by the principal investigator (PG), with the support of two independent researchers (RS and TM), based on the selection and summary of the main points of the extraction.

The risk of bias assessment was conducted using the adapted version of the Effective Public Health Practice Project Quality Assessment tool (EPHPP),^16^ which allows the assessment of five methodological domains of a community-based intervention study: (A) selection, (B) blinding of the researchers who collected and analyzed the data, (C) methods used to collect the data, (D) losses and/or dropouts and (E) analysis. This adapted version of the EPHPP and an explanatory text on how the instrument was used can also be obtained by contacting the corresponding author.

## RESULTS

**Figure 1:**
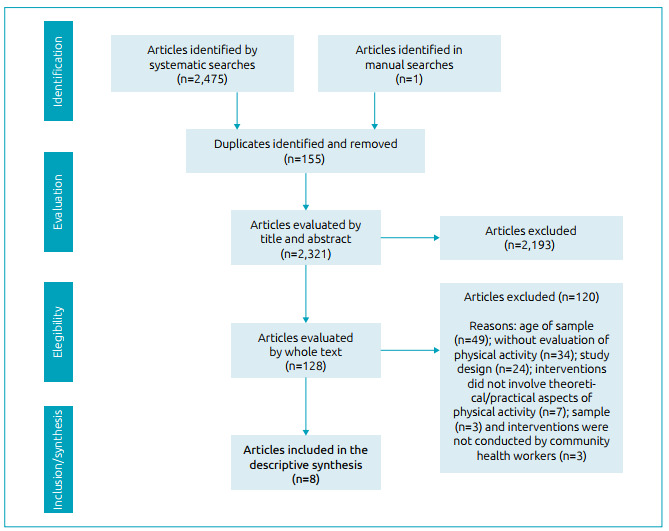
Flowchart of systematic review.

The flowchart (Figure 1) details the numbers and phases of the systematic review. After identifying and removing duplicates (n=155), 2,321 studies were evaluated by their titles and abstracts. Of these, 128 were referred for evaluation by their full texts, of which 120 were excluded, with the following reasons: sample age (n=49); non-assessment of PA (n=34); study design (n=24); interventions that did not involve theoretical/practical aspects of PA (n=7); sample (n=3); interventions that were not conducted by CHW (n=3). Thus, the descriptive synthesis of the present review was composed of data from eight original studies.^17^ ^,^ ^18^ ^,^ ^19^ ^,^ ^20^ ^,^ ^21^ ^,^ ^22^ ^,^ ^23^ ^,^ ^24^

Most of the interventions were carried out in the USA (n=7; 87.5%),^17^ ^,^ ^18^ ^,^ ^19^ ^,^ ^20^ ^,^ ^21^ ^,^ ^22^ ^,^ ^24^ covering children and adolescents between 4^22^ and 15^19^ ^,^ ^20^ years. The samples varied between 15^20^ and 2,965^23^ participants, with girls representing between 41^22^ and 73%^17^ (Table 1). There was great variability in the primary outcomes of the interventions, with emphasis on healthy eating and promotion of PA,^18^ ^,^ ^19^ ^,^ ^21^ ^,^ ^24^ as well as in the target populations, with a greater focus on immigrants,^18^ ^,^ ^22^ ^,^ ^23^ ^,^ ^24^ low-income communities^21^ ^,^ ^23^ and NCD patients.^17^ ^,^ ^19^ ^,^ ^20^

**Table 1:** Descriptive characteristics of the studies included (n=8).

Reference	Country	Mean age	Sample (% girls)	Sample characteristics
Burnet et al.^17^	USA	11	30 (73)	Blacks, with overweight and family history of DM2
Crespo et al.^18^	USA	6	808* (50)	Latino children
Shaibi et al.^20^	USA	15	15 (nd)	Obese Latinos
Rieder et al.^19^	USA	15	91 (54)**	Low-income obese
Subtirelu et al.^21^	USA	13	25 (60)	Low-income (predominantly Latinos).
Ross et al.^22^	USA	4	49 (41)	Latinos
Waters et al.^23^	AUS	5-12***	2,965 (nd)	Low-income
Wieland et al.^24^	USA	13	81 (52)	Latinos, Somalis and Sudanese

*number of parents and their respective children; **percent of girls in the initial sample of the cohort (n=349); ***age range; AUS: Australia; DM2: type 2 diabetes mellitus; USA: United States of America; nd: not described.

In five interventions, the participation of CHW with ethnic characteristics similar to those of the population of interest was noted,^17^ ^,^ ^18^ ^,^ ^20^ ^,^ ^22^ ^,^ ^24^ facilitating the implementation of bilingual and bicultural actions.^18^ ^,^ ^20^ ^,^ ^22^ ^,^ ^24^ In all studies previous training of CHW was carried out to better implement educational activities. By study design, the synthesis was composed of five non-controlled trials^17^ ^,^ ^19^ ^,^ ^20^ ^,^ ^21^ ^,^ ^22^ and three randomized controlled trials,^18^ ^,^ ^23^ ^,^ ^24^ with interventions that were implemented between 2.5 and 41 months. Along the same line, there was great variation in the strategies adopted, highlighting the leadership or support of the CHW in educational activities, practices, home visits, support by phone calls, involvement of parents/guardians and approaches that took the contextual situation of the children and adolescents into account (Chart 1).

**Chart 1:** Synthesis of interventions (n=8).

Burnet et al.^17^ (Type of study: NCT; Duration: 3.5 months) Five trained black workers who conducted the educational sessions with the families. Protocol: 14 weekly sessions of behavioral activities and alternating knowledge about PA and healthy nutrition, with weekly goals
Crespo et al.^18^ (Type of study: RCT; Duration: 12 months) Trained, bilingual and bicultural promoting workers who led educational activities. Protocol: educational measures in the family context (seven home visits and four phone calls) and community context (actions in the physical structures, support for theoretical/practical actions of the teachers).
Shaibi et al.^20^ (Type of study: NCT; Duration: 12 months) Trained, bilingual and bicultural promoting workers, who conducted educational sessions. Protocol: 12 educational sessions and three weekly sessions of PA (60 minutes each).
Rieder et al.^19^ (Type of study: NCT; Duration: 9 months) Trained youth leaders who acted in support of the educational measures and measures of maintenance and engagement of the participants. Protocol: 12 weeks of intervention and 6 months of maintenance, with objectives and monitoring. Educational health activities and PA practices (1-4 times a week). Monthly activities with families.
Subtirelu et al.^21^ (Type of study: NCT; Duration: 9 months) Trained CHW who guided entry into local programs, also offering advice and ongoing evaluation on PA and healthy eating. Protocol: guidance for participating in local PA and/or nutrition programs. Individualized approach, considering personal and contextual factors.
Ross et al.^22^ (Type of study: NCT; Duration: 2.5 months) Nine trained promoting workers, bilingual and bicultural, who developed educational actions, practices and problem-solving at home visits. Protocol: 10 home visits to families (90 minutes), with educations activities, practices and problem-solving related to eating portions of fruits and vegetables, reducing TV time, promoting PA, less consumption of sugary drinks and greater consumption of water.
Waters et al.^23^ (Type of study: RCT; Duration: 41 months) Trained community development workers who conducted educational actions, providing information and guiding the personalized development of intervention strategies. Protocol: the school community determined the content of the intervention strategies on the basis of evidence of success in relation of the indicators evaluated, the development of sustainable changes in schools, homes and community environments and in contextual and programmatic points of the interventions, and their impacts on the results as well.
Wieland et al.^24^ (Type of study: RCT; Duration: 24 months) Family health promoters, bilingual and trained, made the home visits and phone calls. Protocol: creation of a manual with 12 modules (themes: healthy eating and PA), implemented in 12 home visits (30-90 minutes) over six months, with 12 phone calls every two weeks during the last six months.

CHW: community health worker; PA: physical activity; RCT: randomized controlled trial; NCT: non-controlled trial.

**Figure 2:**
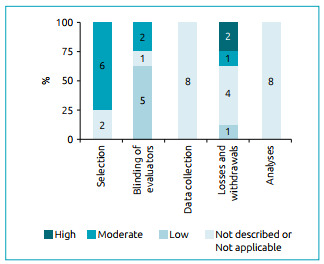
Analysis of risk of bias.

As for the risk of bias, it was observed that all studies were classified as low risk of bias in the domains methods used for data collection and analysis. As for losses and/or dropouts, half of the studies had a low risk of bias.^20^ ^,^ ^22^ ^,^ ^23^ ^,^ ^24^ In the selection domain, six studies were classified as high risk of bias due to the socioeconomic, ethnic and clinical specificities of the samples, allowing for less generalization of the findings.^17^ ^,^ ^18^ ^,^ ^19^ ^,^ ^20^ ^,^ ^22^ ^,^ ^24^ Regarding the blinding domain of the researchers who collected and analyzed the data, no information was provided in five studies (Figure 2).^17^ ^,^ ^19^ ^,^ ^20^ ^,^ ^21^ ^,^ ^22^

Regarding the instruments used to assess PA, questionnaires were used in six interventions and accelerometers in two (Chart 2). Even though PA was evaluated using different indicators, significant results were identified in three non-controlled trials: reduction of daily blocks of 30 minutes of physical inactivity (-26.8%),^20^ increase in the number of individuals involved in vigorous PA for at least six days in the last two weeks (12.4%)^19^ and an increase in the number of adolescents who reported moderate and vigorous PA for at least 60 minutes/day (2.4).^21^ On the other hand, it is worth mentioning the non-significant results of the randomized controlled trials^18^ ^,^ ^23^ ^,^ ^24^ and the studies that used objective measures (accelerometers) for the PA levels.^23^ ^,^ ^24^

**Chart 2:** Instruments and results relative to physical activity indicators (n=8).

**Randomized controlled trials**
Crespo et al.^18^ (QDS) Starting with the PA indicator compared to the other children, changes of 0.36 were seen in the famíly+community group and of 0.05 in the control group, considering the data between post-intervention and baseline (without statistical significance).
Waters et al.^23^ (Two QDS: one for parents/guardians and other for children). There was no effect of intervention on self-reported levels of PA (numerical information not shown).
Wieland et al.^24^ (Kinetic Activity Monitor accelerometer) There were no statistically significant differences between the groups at 6 (changes in MVPA: intervention: 12 min/d and control: 9.3 min/d) and 12 (changes in MVPA: intervention: -4.3 min/d and control: -16.2 min/d) months.
**Non-controlled trials**
Burnet et al.^17^ (Questionnaire used in Aaron et al.*) Analysis of 4 months: (I) >20 min of VPA in ≥3 d of the last week: from 46% (n=13) to 58% (n=14); (II) >20 min of light PA in ≥3 d of the last week: from 39% (n=11) to 50% (n=12) and (III) ≥2 h/d of walking: from 26% (n=7) to 58% (n=14). No statistically significant difference.
Shaibi et al.^20^ (3-day Physical Activity Recall - 3DPAR) Significant difference in blocks of 30 min/d of physical inactivity: from 15.7 to 11.5 (change of -26.8%). No differences in levels of MVPA (blocks of 30 min/d): from 2.3 to 2.9 (change of 26.1%).
Rieder et al.^19^ (Modifiable Activity Questionnaire for Adolescents) Significant increase in VPA ≥6 d/2 weeks: from 13.2 to 25.6%. Changes were not significant in variables MPA (1-5 d and ≥6 d/2 weeks) and PA (1-5 d/2 weeks).
Subtirelu et al.^21^ (QDS) The comparison of assessments of baseline revealed significant changes in levels of 60 min/d of MVPA (from 3.3 to 5.7; p<0.001).
Ross et al.^22^ (ActiGraph GT3X accelerometer). No PA indicator showed statistically significant differences between baseline and end of the intervention: very light PA (change: -0.2); light PA (change: 0.2), MPA (change: 0.1), VPA (change: -0.2) and total (change: -0.1).

*Aaron et al.^25^; PA: physical activity; MPA: moderate physical activity; MVPA: moderate and vigorous physical activity; VPA: vigorous physical activity; d: day(s); h: hour(s); min: minute(s); QDS: questionnaire developed for the study.

Likewise, and regardless of the high variability observed between the SB indicators analyzed by the original studies (Chart 3), two positive results can be highlighted: reduction in the number of daily blocks of 30 minutes of screen time (-46.4%)^20^ and reduced SB during the weekend (-0.9 ­hours/­day).^21^ Also, as seen in the PA indicators, no positive results were observed in randomized controlled trials^18^ ^,^ ^22^ ^,^ ^24^ and in studies that evaluated SB by means of accelerometers.^22^ ^,^ ^24^

**Chart 3:** Instruments and results relative to the indicators of sedentary behavior (n=8).

**Randomized controlled trials**
Crespo et al.^18^ (QDS) Indicator of watching TV while preparing for school: change of -0.22 famíly+community group and -0.09 in control group, comparing data between 1 year after the end of the intervention and baseline (no statistical significance).
Waters et al.^23^ (Two QDS: one for parents/guardians and other for children). There was no effect of intervention on self-report levels of SB (numerical information not shown).
Wieland et al.^24^ (Kinetic Activity Monitor accelerometer). No statistically significant differences were found at 6 (intervention changes: -11.7 min/d and control: -1.6 min/d) and 12 (intervention changes: 55.5 min/d and control: 73.5 min/d) months between the intervention and control groups for SB.
**Non-controlled trials**
Burnet et al.^17^ (Questionnaire used in Aaron et al.*). Hours watching TV: from 3.2 to 4.3, not significant.
Shaibi et al.^20^ (3-day Physical Activity Recall - 3DPAR). Significant difference in daily indicator blocks of 30 min of TV time: from 5.6 to 3.0 (change of -46.4%).
Rieder et al.^19^ (Modifiable Activity Questionnaire for Adolescents). Hours of TV, computer and videogame: 2-5 h/d (from 49 to 48%); ≥ 6 h/d (from 35 to 27%), no statistically significant difference between the measures.
Subtirelu et al.^21^(QDS) The comparisons of assessments of baseline revealed a significant difference in SB during the weekend (baseline 3.5 h/d vs. follow-up 2.6 h/d, p<0,001). No differences between SB on weekdays (from 4.5 h/d to 2.1 h/d).
Ross et al.^22^ (ActiGraph GT3X accelerometer) Change of 0.1 min/h in SB, not significant.

*Aaron et al.^25^; SB: sedentary behavior; d: day(s); h: hour(s); min: minute(s); QDS: questionnaire developed for the study.

## DISCUSSION

On the basis of the results of eight interventions conducted by CHW with the purpose of promoting PA in children and adolescents, we found the main findings to be: (i) differences in increasing PA levels, considering that significant changes were found in only three non-controlled trials, assessed by questionnaires; (ii) most of the strategies were directed at specific groups of vulnerable adolescents (considering ethnic, economic and/or NCD diagnosis factors), highlighting the importance of the action of CHW in the context of communities; (iii) the role of CHW in implementing and supporting educational actions.

More specifically, of the three results that suggest an increase in PA levels,^19^ ^,^ ^20^ ^,^ ^21^ it is worth mentioning interventions lasting between 9^19^ ^,^ ^21^ and 12 months,^20^ aimed at low-income obese Latino populations. Regarding the protocols, we can point out the interventions that combined theoretical and practical activities.^19^ ^,^ ^20^

These findings corroborate the evidence from a previous study, which reinforced the recommendation that aspects related to PA should be prioritized in interventions aimed at populations of children and adolescents in economically disadvantaged contexts.^26^ In this perspective, it is important that more efforts be made to studying the operational processes that are best for increasing PA levels and reducing SB, so that these impacts on the health indicators of these populations can also be assessed.

In general, a good part of the interventions incorporated activities for the parents, aiming at effects on the family as a whole. On the other hand, there was limited intersectoral dialogue, so that the actions of CHW can be more articulated with the work of school teams, to strengthen the relationships of educational work in communities and health work in schools. Future studies may take this point into account, since school interventions are potential,^27^ with particular reference to the contexts of economically disadvantaged populations.^26^

In view of the work developed in the communities in which they reside (or in communities that have socioeconomic, ethnic and/or cultural proximity), the care actions directed to specific groups of greater vulnerability seem to be a potential element of the work of the CHW, since they take into account the specificities and contexts of local populations, as well as favoring the establishment of links between other professionals working in the health system and the community. For these reasons, the CHW connection strategy seems to be especially relevant in these situations, highlighting the evidence from other reviews that addressed other health outcomes.^9^ ^,^ ^10^ ^,^ ^28^

Since the studies included were conducted in high-income countries but aimed at populations at greatest risk, the importance of introducing CHW in non-universal health systems is reinforced. Even recognizing the important role played by the CHW in the interventions included, there is no indication that the actions taken are continuous in these populations, which highlights the implementation of permanent and procedural actions in these contexts of greater vulnerability. Accordingly, it is possible to raise the hypothesis that the introduction of CHW in health systems, acting in a longitudinal way, may impact the health situation of the most vulnerable populations.

In the case of the United States, more specifically, where seven of the eight included interventions occurred, the present synthesis corroborates the findings reported by Perry et al.,^8^ in which CHW efforts to reduce the burden of morbidity and mortality are discussed, especially in situations of NCD in more vulnerable populations, reinforcing their importance in community services and primary health care teams. The CHW strategy articulated with other actions and policies that aim to expand access and use of health services, such as the Affordable Care Act, can contribute to mitigate the gap between high- and low-income Americans with regard to life expectancy and other health indicators.^29^

Thus, when considering the performance potential of CHW in these most vulnerable communities, highlighting the bilingual and bicultural understanding, it is possible to estimate the increase in the possibility of articulation between the people served and the health team, to favor the most appropriate referrals and, consequently, the resolution of cases. Therefore, its liaison role between the health service and the communities is strengthened by its capacity for translation, cultural mediation and facilitation of health actions in different countries.^30^ ^,^ ^31^

Even with the heterogeneity observed in the primary outcomes of the interventions, most strategies were directed towards healthy eating and PA, largely because they fit as modifiable behaviors for the control of a series of NCD, such as overweight/obesity and type 2 diabetes mellitus, very present in the populations covered by this synthesis. Thus, it is worth noting that all interventions included presented educational strategies and, in some of them, directions not just restricted to PA but also involving a broader view of health, with the dissemination of information, practical activities, changes in environments and actions for problem-solving, with a particular focus on healthy eating and weight management. A previous review also pointed out the role of CHW in implementing educational interventions on the themes of food security and immunizations in groups of children and adolescents.^9^

Recognizing the importance of CHW in contexts where there is a shortage of health professionals, it is important that future studies reinforce the processes of recruiting, supervising, encouraging and offering equipment for actions, knowing that these issues can improve their performance.^32^ In addition, it is worth mentioning the importance of evaluating these interventions beyond the concept of effectiveness, involving, for example, the processes of adoption, scope, implementation^33^ and cost-effectiveness,^34^ so that the results produced at the PA levels could be more well contextualized to the potentials and limitations observed throughout the intervention process,^33^ ^,^ ^35^ as well as the economic impacts of these actions on the health system. In a large part of the included studies, it was only pointed out that the CHW received previous training for the implementation of the intervention, without further details on themes and actions, supervision and problem-solving throughout the process or better information on the resources and equipment that were used.

In view of the characteristics of the studies that comprised the present review, it can be suggested to carry out interventions in low- and middle-income countries and/or in countries that have CHW in their respective health systems, as is the case in Brazil, where it is registered its importance, for example, in actions aimed at controlling the weight of children, breastfeeding and late introduction of the bottle.^36^ Also, proposals for permanent health education directed to CHW can be offered in a continuous and longitudinal way on the part of health systems, so that new CHW can also receive adequate knowledge and articulate with local health demands that enable their participation in a more comprehensive perspective.^37^

In the same sense, it can be suggested that this training addresses the fundamental mechanisms that lead to changes in PA in children and adolescents, aiming to expand and improve PA opportunities throughout the day, as suggested by the theory of Beets et al.^38^ In addition, this training can enable CHW to apply various tools for assessing PA and other aspects, such as quality of life and eating habits, since children and adolescents may be exposed to various health risk behaviors.^39^

On the basis of the analysis of the risk of bias, it can be highlighted that the high heterogeneity among the populations selected for the interventions represents one of the main limiting factors of the present review, since the contextual specificities and the profiles of the participants reduce the ability to generalize the evidence. In addition, limitations can also be pointed out with regard to the design of the included interventions, since five of them did not have comparison groups, and regarding losses and dropouts during the follow-up, in which only half of the studies were classified as low risk. As for the instruments and procedures adopted in data collection, even though all studies were classified as low risk of bias, it is worth pointing out their high heterogeneity, suggesting greater caution when comparing and extrapolating the results.

Finally, even though most of the interventions included did not have significant effects on increasing PA levels in children and adolescents, the available evidence points to the importance of the work of CHW in the contexts of greater vulnerability. It is also possible to suggest the development of interventions conducted by CHW in schools, in partnership with the school team and supported by evaluations of the implementation processes and cost-effectiveness.
